# The effect of changing ventilator settings on indices of ventilation inhomogeneity in small ventilated lungs

**DOI:** 10.1186/1471-2466-6-20

**Published:** 2006-08-18

**Authors:** G Schmalisch, H Proquitté, CC Roehr, RR Wauer

**Affiliations:** 1 Clinic of Neonatology, Charité Universitätsmedizin Berlin, Berlin, Germany

## Abstract

**Background:**

In ventilated newborns the use of multiple breath washout (MBW) techniques for measuring both lung volume and ventilation inhomogeneity (VI) is hampered by the comparatively high dead space fraction. We studied how changes in ventilator settings affected VI indices in this particular population.

**Methods:**

Using a computer simulation of a uniformly ventilated volume the interaction between VI indices (lung clearance index (LCI), moment ratios (M_1_/M_0_, M_2_/M_0_, AMDN_1_, AMDN_2_) of the washout curve) and tidal volume (V_T_), dead space (V_D_) and functional residual capacity (FRC) were calculated. The theoretical results were compared with measurements in 15 ventilated piglets (age <12 h, median weight 1135 g) by increasing the peak inspiratory pressure (PIP). FRC and VI indices were measured by MBW using 0.8% heptafluoropropane as tracer gas.

**Results:**

The computer simulation showed that the sensitivity of most VI indices to changes in V_D_/V_T _and V_T_/FRC increase, in particular for V_D_/V_T _> 0.5. In piglets, the raised PIP caused a significant increase of V_T _from 15.4 ± 9.5 to 21.9 ± 14.7 (p = 0.003) and of the FRC from 31.6 ± 14.7 mL to 35.0 ± 15.9 mL (p = 0.006), whereas LCI (9.15 ± 0.75 to 8.55 ± 0.74, p = 0.019) and the moment ratios M_1_/M_0_, M_2_/M_0 _(p < 0.02) decreased significantly. No significant changes were seen in AMDN_1 _and AMDN_2_. The within-subject variability of the VI indices (coefficient of variation in brackets) was distinctly higher (LCI (9.8%), M_1_/M_0 _(6.6%), M_2_/M_0 _(14.6%), AMDN_1 _(9.1%), AMDN_2 _(16.3%)) compared to FRC measurements (5.6%). Computer simulations showed that significant changes in VI indices were exclusively caused by changes in V_T _and FRC and not by an improvement of the homogeneity of alveolar ventilation.

**Conclusion:**

In small ventilated lungs with a high dead space fraction, indices of VI may be misinterpreted if the changes in ventilator settings are not considered. Computer simulations can help to prevent this misinterpretation.

## Background

In ventilated newborns respiratory problems are often caused by impaired lung development and uneven alveolar ventilation. Surfactant deficiency or dysfunction may increase the ventilatory inhomogeneity by collapse or over distention of the alveoli. Thus, there is an increasing clinical interest in multiple breath washout techniques (MBW) to measure both the functional residual capacity (FRC) and ventilatory inhomogeneity (VI) indices [1-4]. Lung clearance index (LCI) and moment ratios of the wash out curve are the most frequently used indices in infancy [5]. Commonly, the first and the second moment related to the zeroth moment (M_1_/M_0_, M_2_/M_0_) are calculated.

Most VI indices are easily calculated, however, a key disadvantage is their dependency on the breathing pattern [6]. In 1975, Saidel et al. [7] suggested that this dependency can be reduced by performing a moment analysis of the washout curve plotted as a function of the cumulative exhaled volume related to the FRC. However, the dependency of the moment ratios on the ventilatory dead space (V_D_) remained. Therefore, Habib and Lutchen [8] replaced the cumulative exhaled volume by the cumulative alveolar volume to reduce the influence of V_D_. They referred to the first two moment ratios of the wash out curve as alveolar-based mean dilution numbers AMDN_1 _and AMDN_2_.

Dead space fractions (V_D _related to the tidal volume V_T_) in adults commonly lie between 0.05 to 0.2 [8]. In ventilated newborns the dead space fraction V_D_/V_T _is often markedly higher [9] depending on the ventilator settings. Typical values lie between 0.4 and 0.6 [10] and in preterm or surfactant-depleted lungs V_D_/V_T _can rise up to 0.7 [11]. Such small lungs are ventilated with a relative low tidal volume to prevent volu-trauma. Mainstream flow sensors and gas analyzers considerably increase the apparatus dead space so that high dead space fractions are not uncommon. The effect of an increased V_D_/V_T _on the sensitivity of VI indices to parameter changes is not well known. Therefore, the aim of this study was to investigate how changing ventilator settings affect the different VI indices in this particular population by mathematical modelling and by measurements in newborn piglets using the MBW technique with heptafluoropropane (HFP) as tracer gas.

## Methods

### Modelling

In patients ventilated with a constant V_T _the LCI is given by

LCI=NLCI⋅VTFRC     (1)
 MathType@MTEF@5@5@+=feaafiart1ev1aaatCvAUfKttLearuWrP9MDH5MBPbIqV92AaeXatLxBI9gBaebbnrfifHhDYfgasaacH8akY=wiFfYdH8Gipec8Eeeu0xXdbba9frFj0=OqFfea0dXdd9vqai=hGuQ8kuc9pgc9s8qqaq=dirpe0xb9q8qiLsFr0=vr0=vr0dc8meaabaqaciaacaGaaeqabaqabeGadaaakeaacqqGmbatcqqGdbWqcqqGjbqscqGH9aqpdaWcaaqaaiabb6eaonaaBaaaleaacqqGmbatcqqGdbWqcqqGjbqsaeqaaOGaeyyXICTaeeOvay1aaSbaaSqaaiabbsfaubqabaaakeaacqqGgbGrcqqGsbGucqqGdbWqaaGaaCzcaiaaxMaadaqadiqaaiabigdaXaGaayjkaiaawMcaaaaa@4191@

where N_LCI _is the number of breaths required to lower the end tidal tracer gas concentration to 1/40^th ^of the starting concentration [12]. The ideal washout curve of an inert gas from a uniformly ventilated volume can be expressed as

cn=c0[11+VTFRC(1−VDVT)]n,n=0,1,2.....N.     (2)
 MathType@MTEF@5@5@+=feaafiart1ev1aaatCvAUfKttLearuWrP9MDH5MBPbIqV92AaeXatLxBI9gBaebbnrfifHhDYfgasaacH8akY=wiFfYdH8Gipec8Eeeu0xXdbba9frFj0=OqFfea0dXdd9vqai=hGuQ8kuc9pgc9s8qqaq=dirpe0xb9q8qiLsFr0=vr0=vr0dc8meaabaqaciaacaGaaeqabaqabeGadaaakeaafaqabeqacaaabaGaee4yam2aaWbaaSqabeaacqqGUbGBaaGccqGH9aqpcqqGJbWydaWgaaWcbaGaeGimaadabeaakmaadmGabaWaaSaaaeaacqaIXaqmaeaacqaIXaqmcqGHRaWkdaWcaaqaaiabbAfawnaaBaaaleaacqqGubavaeqaaaGcbaGaeeOrayKaeeOuaiLaee4qameaamaabmGabaGaeGymaeJaeyOeI0YaaSaaaeaacqqGwbGvdaWgaaWcbaGaeeiraqeabeaaaOqaaiabbAfawnaaBaaaleaacqqGubavaeqaaaaaaOGaayjkaiaawMcaaaaaaiaawUfacaGLDbaadaahaaWcbeqaaiabb6gaUbaakiabcYcaSaqaaiabb6gaUjabg2da9iabicdaWiabcYcaSiabigdaXiabcYcaSiabikdaYiabc6caUiabc6caUiabc6caUiabc6caUiabc6caUiabb6eaojabc6caUaaacaWLjaGaaCzcamaabmGabaGaeGOmaidacaGLOaGaayzkaaaaaa@5A07@

where c^n ^is the end-expiratory gas concentration of the n^th ^breathing cycle and c_0 _is the initial gas concentration. Using computer simulations of the washout curve LCI can be calculated as a function of V_T_/FRC and V_D_/V_T_.

The moments M_0_, M_1_, M_2 _of the washout curve were calculated up to N_LCI_. For a constant V_T _the moment ratios are given by

M1/M0=(VTFRC)∑i=0NLCIi⋅ci∑i=0NLCIciandM2/M0=(VTFRC)2∑i=0NLCIi2⋅ci∑i=0NLCIci,     (3)
 MathType@MTEF@5@5@+=feaafiart1ev1aaatCvAUfKttLearuWrP9MDH5MBPbIqV92AaeXatLxBI9gBaebbnrfifHhDYfgasaacH8akY=wiFfYdH8Gipec8Eeeu0xXdbba9frFj0=OqFfea0dXdd9vqai=hGuQ8kuc9pgc9s8qqaq=dirpe0xb9q8qiLsFr0=vr0=vr0dc8meaabaqaciaacaGaaeqabaqabeGadaaakeaafaqabeqadaaabaGaeeyta00aaSbaaSqaaiabigdaXaqabaGccqGGVaWlcqqGnbqtdaWgaaWcbaGaeGimaadabeaakiabg2da9maabmGabaWaaSaaaeaacqqGwbGvdaWgaaWcbaGaeeivaqfabeaaaOqaaiabbAeagjabbkfasjabboeadbaaaiaawIcacaGLPaaadaWcaaqaamaaqahabaGaemyAaKMaeyyXICTaem4yam2aaWbaaSqabeaacqWGPbqAaaaabaGaemyAaKMaeyypa0JaeGimaadabaGaemOta40aaSbaaWqaaiabdYeamjabdoeadjabdMeajbqabaaaniabggHiLdaakeaadaaeWbqaaiabdogaJnaaCaaaleqabaGaemyAaKgaaaqaaiabdMgaPjabg2da9iabicdaWaqaaiabd6eaonaaBaaameaacqWGmbatcqWGdbWqcqWGjbqsaeqaaaqdcqGHris5aaaaaOqaaiabbggaHjabb6gaUjabbsgaKbqaaiabb2eannaaBaaaleaacqaIYaGmaeqaaOGaei4la8Iaeeyta00aaSbaaSqaaiabicdaWaqabaGccqGH9aqpdaqadiqaamaalaaabaGaeeOvay1aaSbaaSqaaiabbsfaubqabaaakeaacqqGgbGrcqqGsbGucqqGdbWqaaaacaGLOaGaayzkaaWaaWbaaSqabeaacqaIYaGmaaGcdaWcaaqaamaaqahabaGaemyAaK2aaWbaaSqabeaacqaIYaGmaaGccqGHflY1cqWGJbWydaahaaWcbeqaaiabdMgaPbaaaeaacqWGPbqAcqGH9aqpcqaIWaamaeaacqWGobGtdaWgaaadbaGaemitaWKaem4qamKaemysaKeabeaaa0GaeyyeIuoaaOqaamaaqahabaGaem4yam2aaWbaaSqabeaacqWGPbqAaaaabaGaemyAaKMaeyypa0JaeGimaadabaGaemOta40aaSbaaWqaaiabdYeamjabdoeadjabdMeajbqabaaaniabggHiLdaaaaaakiabcYcaSiaaxMaacaWLjaWaaeWaceaacqaIZaWmaiaawIcacaGLPaaaaaa@8F13@

where "i" is the number of the breathing cycle. For an infinite number of cycles M_1_/M_0 _has a fixed limit given by

M1/M0=VTVT−VD.     (4)
 MathType@MTEF@5@5@+=feaafiart1ev1aaatCvAUfKttLearuWrP9MDH5MBPbIqV92AaeXatLxBI9gBaebbnrfifHhDYfgasaacH8akY=wiFfYdH8Gipec8Eeeu0xXdbba9frFj0=OqFfea0dXdd9vqai=hGuQ8kuc9pgc9s8qqaq=dirpe0xb9q8qiLsFr0=vr0=vr0dc8meaabaqaciaacaGaaeqabaqabeGadaaakeaacqqGnbqtdaWgaaWcbaGaeGymaedabeaakiabc+caViabb2eannaaBaaaleaacqaIWaamaeqaaOGaeyypa0ZaaSaaaeaacqqGwbGvdaWgaaWcbaGaeeivaqfabeaaaOqaaiabbAfawnaaBaaaleaacqqGubavaeqaaOGaeyOeI0IaeeOvay1aaSbaaSqaaiabbseaebqabaaaaOGaeiOla4IaaCzcaiaaxMaadaqadiqaaiabisda0aGaayjkaiaawMcaaaaa@4072@

Following Habib and Lutchen [8], the replacement of V_T _by the alveolar ventilation V_T_-V_D _yield the alveolar-based mean dilution numbers

AMDN1=(VT−VDFRC)∑i=0NLCIi⋅ci∑i=0NLCIciAMDN2=(VT−VDFRC)2∑i=0NLCIi2⋅ci∑i=0NLCIci     (5)
 MathType@MTEF@5@5@+=feaafiart1ev1aaatCvAUfKttLearuWrP9MDH5MBPbIqV92AaeXatLxBI9gBaebbnrfifHhDYfgasaacH8akY=wiFfYdH8Gipec8Eeeu0xXdbba9frFj0=OqFfea0dXdd9vqai=hGuQ8kuc9pgc9s8qqaq=dirpe0xb9q8qiLsFr0=vr0=vr0dc8meaabaqaciaacaGaaeqabaqabeGadaaakeaafaqabeqacaaabaGaeeyqaeKaeeyta0KaeeiraqKaeeOta40aaSbaaSqaaiabigdaXaqabaGccqGH9aqpdaqadiqaamaalaaabaGaeeOvay1aaSbaaSqaaiabbsfaubqabaGccqGHsislcqqGwbGvdaWgaaWcbaGaeeiraqeabeaaaOqaaiabbAeagjabbkfasjabboeadbaaaiaawIcacaGLPaaadaWcaaqaamaaqahabaGaemyAaKMaeyyXICTaem4yam2aaWbaaSqabeaacqWGPbqAaaaabaGaemyAaKMaeyypa0JaeGimaadabaGaemOta40aaSbaaWqaaiabdYeamjabdoeadjabdMeajbqabaaaniabggHiLdaakeaadaaeWbqaaiabdogaJnaaCaaaleqabaGaemyAaKgaaaqaaiabdMgaPjabg2da9iabicdaWaqaaiabd6eaonaaBaaameaacqWGmbatcqWGdbWqcqWGjbqsaeqaaaqdcqGHris5aaaaaOqaaiabbgeabjabb2eanjabbseaejabb6eaonaaBaaaleaacqaIYaGmaeqaaOGaeyypa0ZaaeWaceaadaWcaaqaaiabbAfawnaaBaaaleaacqqGubavaeqaaOGaeyOeI0IaeeOvay1aaSbaaSqaaiabbseaebqabaaakeaacqqGgbGrcqqGsbGucqqGdbWqaaaacaGLOaGaayzkaaWaaWbaaSqabeaacqaIYaGmaaGcdaWcaaqaamaaqahabaGaemyAaK2aaWbaaSqabeaacqaIYaGmaaGccqGHflY1cqWGJbWydaahaaWcbeqaaiabdMgaPbaaaeaacqWGPbqAcqGH9aqpcqaIWaamaeaacqWGobGtdaWgaaadbaGaemitaWKaem4qamKaemysaKeabeaaa0GaeyyeIuoaaOqaamaaqahabaGaem4yam2aaWbaaSqabeaacqWGPbqAaaaabaGaemyAaKMaeyypa0JaeGimaadabaGaemOta40aaSbaaWqaaiabdYeamjabdoeadjabdMeajbqabaaaniabggHiLdaaaOGaaCzcaiaaxMaadaqadiqaaiabiwda1aGaayjkaiaawMcaaaaaaaa@9124@

For a well-mixed volume and an infinite number of breathing cycles, AMDN_1 _is equal one regardless of V_D_/V_T _or V_T_/FRC

AMDN1=VT−VDVT−VD=1     (6)
 MathType@MTEF@5@5@+=feaafiart1ev1aaatCvAUfKttLearuWrP9MDH5MBPbIqV92AaeXatLxBI9gBaebbnrfifHhDYfgasaacH8akY=wiFfYdH8Gipec8Eeeu0xXdbba9frFj0=OqFfea0dXdd9vqai=hGuQ8kuc9pgc9s8qqaq=dirpe0xb9q8qiLsFr0=vr0=vr0dc8meaabaqaciaacaGaaeqabaqabeGadaaakeaacqqGbbqqcqqGnbqtcqqGebarcqqGobGtdaWgaaWcbaGaeGymaedabeaakiabg2da9maalaaabaGaeeOvay1aaSbaaSqaaiabbsfaubqabaGccqGHsislcqqGwbGvdaWgaaWcbaGaeeiraqeabeaaaOqaaiabbAfawnaaBaaaleaacqqGubavaeqaaOGaeyOeI0IaeeOvay1aaSbaaSqaaiabbseaebqabaaaaOGaeyypa0JaeGymaeJaaCzcaiaaxMaadaqadiqaaiabiAda2aGaayjkaiaawMcaaaaa@44FD@

and an AMDN_1 _> 1 implies inhomogeneity purely at alveolar level [8]. For very low dead spaces (V_D_/V_T_≈0) the alveolar-based mean dilution numbers are equal the moment ratios (AMDN_1 _= M_1_/M_0_, AMDN_2 _= M_2_/M_0_). An important feature of all VI indices is that they rise with increasing inhomogeneity of the alveolar ventilation which can be shown easily by computer simulations of multi-compartment models.

### Animal experiments

Fifteen newborn piglets (age <12 h, median weight 1135 g) placed in supine position within a heated incubator were anesthetized (azaperon 8 mg·kg^-1 ^and ketamin 10 mg·kg^-1^), intubated (shortened neonatal endotracheal tube (ETT) with 3.5 mm outer diameter, Vygon, Ecouen, France), paralyzed (pancuronium-bromide 0.2 mg·kg^-1^·hour^-1^) and mechanically ventilated with a neonatal ventilator (Babylog 8000, Draeger, Lübeck, Germany). During the study period, air flow (6 L/min), respiratory rate of 40/min and fraction of inspired oxygen (FiO_2_) of 1.0 were kept constant. Positive end-expiratory pressure (PEEP) was set to zero, peak inflation pressure (PIP) was set initially to 8.3 ± 3.1 cmH_2_O and elevated to 12.1 ± 5.0 cmH_2_O. Ventilatory parameters were taken from the Babylog 8000 and recorded continuously. Lung volume and VI indices were measured by heptafluoropropane (HFP) wash in and wash out as previously described [13]. Briefly, a new infrared HFP sensor was sited between the flow sensor of the Babylog 8000 and the ETT. The total apparatus dead space of HFP sensor, flow sensor and ETT was 4.5 mL determined by water displacement. The constant flow of the ventilator was 8 L/min in all measurements. Using a mechanical valve to start wash in or wash out, a HFP flow from a gas cylinder (medical grade HFP, Solvay, Hannover, Germany) was fed into the inspiratory limb of the ventilatory circuit to achieve a constant HFP concentration of 0.8%. The flow signal of the Babylog 8000 and the concentration signal from the HFP sensor were used to calculate FRC and the VI indices from the wash-in or wash-out curve up to 1/40^th ^of the starting concentration by an external computer. The Fowler dead space V_D _was determined from the first 5 cycles. The calculation was stopped automatically after N cycles when the total amount of alveolar turnovers exceed the tenfold of the calculated FRC (minimum number 40 cycles).

After instrumentation and onset of mechanical ventilation a stabilisation period of 15 minutes was allowed before the measurements were started with a HFP wash-in procedure (FRC_wash-in_) and a consecutive wash-out procedure (FRC_wash-out_). Such a cycle was accepted for evaluation if the deviation between FRC_wash-in _and FRC_wash-out _was lower than 20% and the V_T _was higher than 4.5 mL (V_Dapp_). In order to investigate the effect of ventilator settings on VI indices PIP was increased by 4 cm H_2_O. After a stabilisation period of 15 minutes the MBW was repeated in the same manner.

### Computer program

A computer program written in Visual Basic (Microsoft Corpor., USA) as a macro of a EXCEL worksheet (Microsoft Office 2000, Microsoft Corpor., USA) was developed to compare the VI indices measured in the piglets with the VI indices of a uniformly ventilated volume using the same ventilator settings [see Additional file 1]. The program calculates the washout curve according equation 2 and the corresponding VI indices according equations 1, 3 and 5.

### Statistics

For each measurement in the piglets at least 5 wash-in and wash-out cycles were performed and averaged. Data are presented as mean ± SD and mean individual differences with 95% CI as appropriate. Differences in the animals were compared by the paired t-test. To assess the within-subject variability of repeated measurements the coefficient of variation (CV) was calculated for all parameters and compared by a rank test. A level of statistical significance of p < 0.05 was accepted.

## Results

### Computer simulation

**Figure 1:**
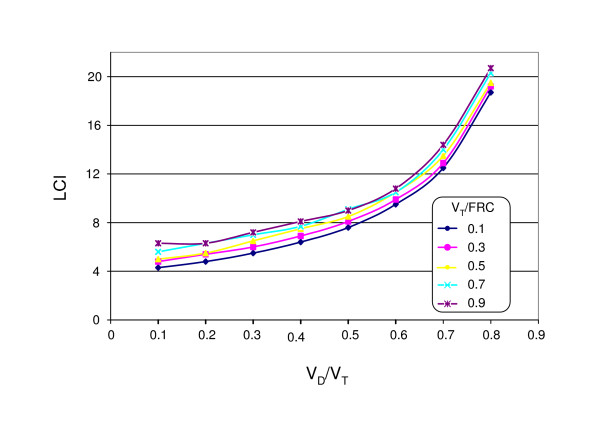
Numerical calculation of the lung clearance index (LCI) of a uniformly ventilated space as function of V_D_/V_T _and V_T_/FRC.

The computer simulation of a uniformly ventilated volume showed that the LCI increased with increasing V_D_/V_T _and V_T_/FRC (Fig. 1). However, the influence of V_D_/V_T _on the LCI is distinctly stronger than that of V_T_/FRC. In particular for V_D_/V_T _> 0.5 the LCI increased dramatically.

**Figure 2:**
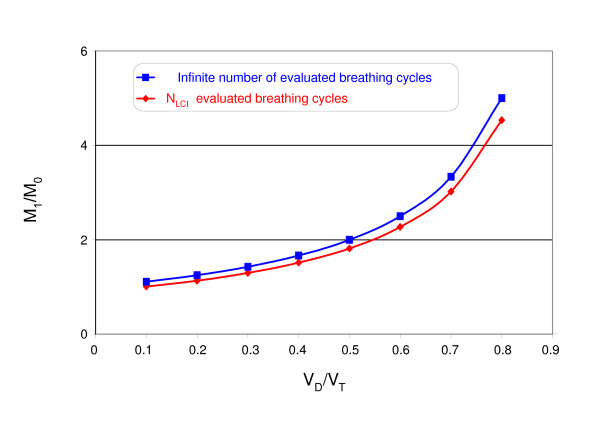
Effect of V_D_/V_T _on the moment ratio M_1_/M_0 _using an infinite number and N_LCI _breathing cycle (The influence of V_T_/FRC on M_1_/M_0 _was negligible).

The moment ratio M_1_/M_0 _was independent of V_T_/FRC but the dependency on V_D_/V_T _remained. As shown in Fig. 2, the calculated values were about 10% lower than predicted by equation 4 because only N_LCI _breathing cycles were evaluated. The second moment ratio M_2_/M_0 _showed a similar dependency on V_D_/V_T _and V_T_/FRC like the LCI.

**Figure 3:**
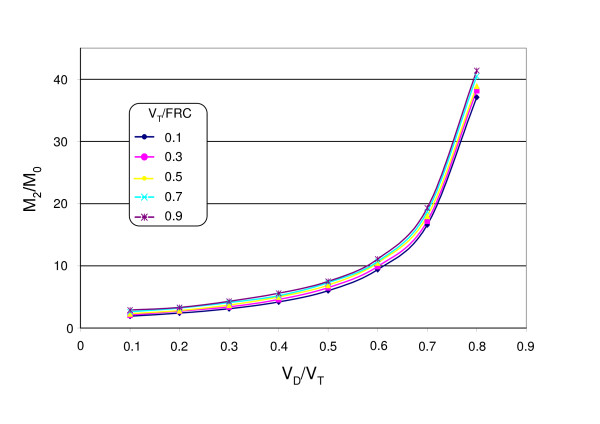
The moment ratio M_2_/M_0 _of an uniformly ventilated space as function of V_D_/V_T _and V_T_/FRC using N_LCI _breathing cycles.

**Figure 4:**
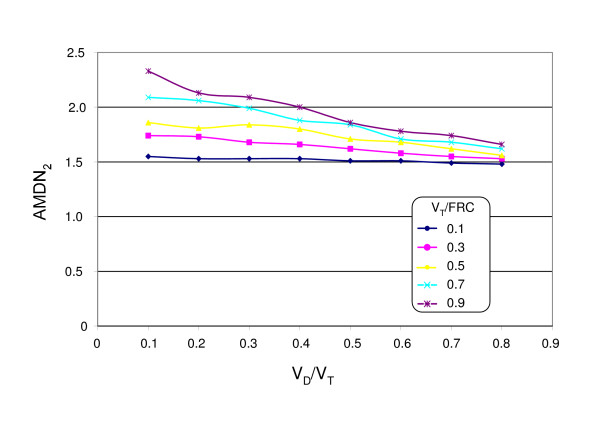
Numerical calculation of the alveolar-based mean dilution numbers AMDN_2_, using N_LCI _breathing cycles.

The alveolar-based mean dilution number AMDN_1 _ranged between 0.91 and 0.94 independent of V_T_/FRC and V_D_/V_T_. Due to the finite number of evaluated cycles AMDN_1 _was <1 as given by equation 6. In contrast to M_2_/M_0 _the dependencies of AMDN_2 _differed considerably and were distinctly lower (Fig. 3). AMDN_2 _decreased with increasing V_D_/V_T _and increased only slightly with increasing V_T_/FRC (Fig. 4).

### Animal study

The results of the FRC and VI measurements in the piglets are shown in Table 1. An increase in PIP of about 4 cmH_2_O caused a significant increase in V_T _of 39% (p = 0.003) and of the FRC of 11% (p = 0.006). Because the increase in V_T _was much higher compared with the increase in the FRC the ratio V_T_/FRC increased significantly (p = 0.003). Due to the increase in V_T _there was a significant decrease of V_D_/V_T _(p = 0.006).

A significant decrease was also seen in the LCI (p = 0.019) and the moment ratios M_1_/M_0 _(p = 0.006) and M_2_/M_0 _(p = 0.017). No significant changes were seen in AMDN_1 _and AMDN_2_.

There was a strong correlation between LCI and the moment ratios (M_1_/M_0_, M_2_/M_0_) with r = 0.885 and r = 0.907, respectively, and independent of which PIP was used. Thus, it was not surprising that there was a similar effect of the increased PIP on LCI and the moment ratios (M_1_/M_0_, M_2_/M_0_). No statistically significant correlations were found between the alveolar-based mean dilution numbers (AMDN_1_, AMDN_2_) and LCI, M_1_/M_0 _and M_2_/M_0_.

The within-subject variability of the measured parameters showed considerable variations but it was not affected by the increase of the PIP. The median CV of all FRC measurements was 5.6%. The median CV of the LCI was significantly greater (9.8%, p = 0.0004). Compared with the LCI the CV of M_1_/M_0 _was significantly smaller (6.6%, p = 0.003), whereas the CV of M_2_/M_0 _was distinctly greater (14.6%, p = 0.004). The CVs of AMDN_1 _(9.1%) and AMDN_2 _(16.3%) were always greater than the CVs of the other moment ratios.

**Figure 5:**
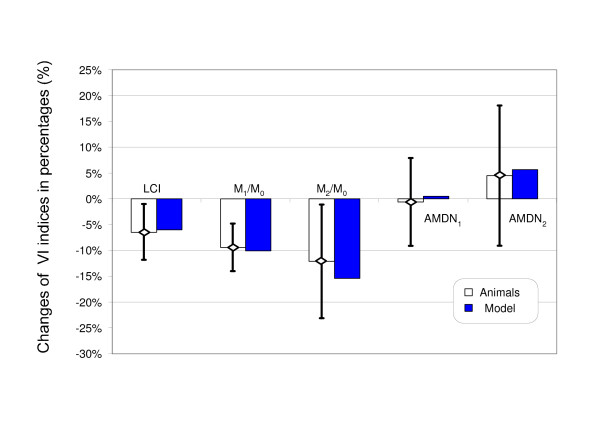
Comparison on the changed VI indices due to increased PIP in the piglets (mean with 95% CI) with the numerically calculated VI indices of a uniformly mixed volume (blue bars) using the same ratios V_D_/V_T _and V_T_/FRC as shown in table 1.

Using the same ratios V_T_/FRC and V_D_/V_T_ as measured in the animals the VI indices of a uniformly ventilated volume were only slightly lower (Table 2) than in the animals measured. The relative changes of the VI indices were in well agreement with the relative changes measured in the animals (Fig. 5). All VI indices calculated for a uniformly ventilated space were within the confidences range of the animal measurements (Fig. 5). This means that the significant changes in the VI indices of Table 1 were exclusively caused by the changes in V_T_/FRC and V_D_/V_T _due to an increase in PIP and not by a more even alveolar ventilation.

## Discussion

The measurement of lung volume and ventilation inhomogeneity by MBW is a fascinating, non-invasive technique. It is relatively easily performed, even in ventilated patients. In a previous study [13], we have shown that by this technique the effect of surfactant-depletion by lung lavage on the FRC and the VI indices is reliably measured: before and after lavage V_D_/V_T _was not significant different, therefore, the significant increase of the VI indices has to be predominantly attributed to the effect of lung lavage. In the present study the measurements were performed in healthy lungs and V_D_/V_T _was distinctly changed by an increase of the PIP. As shown in Fig. 5 the changes of the VI indices are mainly caused by physical laws of gas mixing.

The interpretation of significant changes in VI indices may be misleading if their dependency on the ventilator settings is not considered. This is a particular problem in small lungs where the relatively high dead space fraction increases the sensitivity of VI indices to parameter changes. Any changes in V_D _(e.g. by applying of a new mainstream sensor) or changes in V_T _and FRC (e.g. by changing of ventilator settings or by surfactant substitution) will affect the VI indices and therefore hamper their comparability.

As shown by the computer simulation, most VI indices increase with increasing V_D_/V_T_. This may explain why in newborns much higher VI indices values were measured [4,13,14] than in spontaneously breathing children [15,16]. These higher values in newborns are more likely an expression of functional dependencies than the result of impaired alveolar ventilation. The relatively good agreement between the VI indices measured in healthy piglets (Table 1) and the calculated VI indices of a uniformly ventilated volume (Table 2) was surprising. There was only a small difference in the VI indices between the animal measurements and the modelling which can be attributed to the more complex ventilation distribution in the lungs of the piglets.

In infancy the LCI is one of most frequently used VI index [16-19] and easy to comprehend. It describes the number of turnovers to lower the end tidal tracer gas concentration to 1/40^th ^of the starting concentration. Theoretically, the LCI is a static value of the flat tail of the washout curve and may vary if the signal is noisy. This explains its relatively high within-subject variability. The limitation on N_LCI _breathing cycle seems to be useful to reduce the influence of the signal noise on the measured LCI. Its main disadvantage is its high dependency on V_T_/V_D _as shown in Fig. 1.

Moment ratios are more abstract mathematical measures considering the whole washout curve. Only for M_1_/M_0 _a theoretical value for a well ventilated volume exists (equation 4). M_1_/M_0 _reflect more the first part of the washout curve, whereas M_2_/M_0 _better describe the tail of the curve. Therefore the within-subject variability of M_2_/M_0 _is distinctly higher compared to M_1_/M_0 _and similar to the within-subject variability of the LCI. A moment analysis makes higher demands on the wash out curve compared to LCI measurements. It requires a rapid rise of the tracer gas after the switch-on so that the full tracers gas concentration is reached during the first inspiratory cycle. This is sometimes difficult to achieve, in particular if the tracer gas is fed into the inspiratory limb of the ventilator circuit, far from the ETT, which may delay such a swift rise. Such a delay is tolerable for FRC measurements but will affect the calculation of the moment ratios.

In contrast to LCI and the ratios M_1_/M_0 _and M_2_/M_0_, no significant effect of the increased PIP on AMDN_1 _and AMDN_2 _was seen, as predicted by the computer simulation. These parameters suggested by Habbib and Lutchen [8] seem indeed to be less sensitive to the changes in the breathing pattern than the other ones. This dos not necessarily mean that they have a higher predictive value: with the exception of the above authors [8], a higher diagnostic value of these corrected moments could not be demonstrated until now [20]. The main problem with these parameters is that they need an exact determination of the Fowler dead space. This is often difficult to evaluate in small lungs because the three phases of the gas concentration-volume diagram of the exhaled air are often not well defined [11]. This may explain why in animals AMDN_1 _and AMDN_2 _often showed a very high within-subject variability (>20%). This high variability may limit their diagnostic value.

A central problem of all moment ratios is their dependency on the number of evaluated breathing cycles [14]. The computer simulation has already shown that the theoretical values for M_1_/M_0 _and AMDN_1 _were not reached due to the finite number of evaluated cycles (Fig. 2). This hampers the comparability of the data between different laboratories if the start and the end of the evaluated breathing cycles are not specified.

The within-subject variability of LCI, M_1_/M_0 _and M_2_/M_0 _in our study was similar to those measured by Shao et al. [14] in preterm infants. In both studies the variability of the VI indices was distinctly higher compared with the CV of the FRC. Thus, in small ventilated lungs the determination of VI indices needs a higher number of wash-in and washout cycles than for FRC measurements to obtain reproducible results.

## Conclusion

With the availability of dead space-minimized mainstream gas analyzers there is an increasing interest to measure ventilation inhomogeneity by MBW techniques. However, the use of VI indices in small lungs needs particular attention. Especially in small ventilated lungs with a relatively high dead space fraction most indices are significantly affected by ventilator settings. Changes in tidal volume and lung volume, or changes in the apparatus dead space hamper their comparison. Model simulations of a uniformly ventilated volume can help to decide if the changes in the VI indices are caused by changing ventilator settings or whether they indicate any changes in the homogeneity of alveolar ventilation.

## Abbreviations

AMDN_1,2 _Alveolar-based mean dilution numbers_1,2_

c^n ^End-expiratory tracer gas concentration of the n^th ^breathing cycle

CV Coefficient of variation

ETT Endotracheal tube

F_I_O_2 _Fraction of inspired oxygen

FRC Functional residual capacity

FRC_wash-in _Functional residual capacity measured by wash-in of the tracer gas

FRC_wash-out _Functional residual capacity measured by wash-out of the tracer gas

HFP Heptafluoropropane

ICU Intensive care unit (for newborn infants)

LCI Lung clearance index

M_1_/M_0 _First-to-zeroth moment ratio

M_2_/M_0 _Second-to-zeroth moment ratio

MBW Multiple breath washout

N Number of breathing cycles

N_LCI _Number of breaths required to lower the tidal tracer gas concentration to 1/40^th ^of the starting concentration

PEEP Positive end-expiratory pressure

PIP Peak inflation pressure

V_D _Deadspace volume

V_Dapp _Apparatus deadspace volume

V_T _Tidal volume

VI Ventilation inhomogeneity

## Competing interests

The author(s) declare that they have no competing interests.

## Authors' contributions

GS and RW had primary responsibility for study design, protocol development, data analysis and writing of the manuscript. HP and CCR carried out all lung volume measurements in the piglets. GS performed all computer simulations and calculations of the indices of ventilation inhomogeneity. All authors read and approved the final manuscript.

## Pre-publication history

The pre-publication history for this paper can be accessed here:



## Supplementary Material

Additional file 1 VI Indices of a well mixed volume. The macro uses the V_D_/V_T _and V_T_/FRC ratios to calculate the tracer gas wash-out curve and the different VI indices of a well mixed volume. Click here for file

## Figures and Tables

**Table 1:** Effect of increased peak inspiratory pressure on ventilation, FRC and ventilatory inhomogeneity indices in 15 ventilated piglets (Presented are groups means ± SD and the mean individual changes with 95%CI compared to the baseline)

	**Baseline**	**Increased peak inspiratory pressure**	**Mean individual difference in % with 95%CI**	**P value**
**V_T _(mL)**	15.4 ± 9.5	21.9 ± 14.7	38.9 (20.4 to 57.45)	**p = 0.003**
**FRC (mL)**	31.6 ± 14.7	35.0 ± 15.9	11.1 (4.5 to 17.8)	**p = 0.006**
**V_T_/FRC**	0.48 ± 0.17	0.59 ± 0.20	25.0 (10.7 to 39.4)	**p = 0.003**
**V_D_/V_T_**	0.49 ± 0.15	0.43 ± 0.13	-10.2 (-21.0 to 0.5)	**p = 0.006**
**LCI**	9.17 ± 0.75	8.55 ± 0.74	-6.4 (-11.8 to -1.0)	**p = 0.019**
**M_1_/M_0_**	2.03 ± 0.16	1.84 ± 0.16	-9.4 (-14.0 to -4.9)	**p = 0.006**
**M_2_M_0_**	8.41 ± 1.60	7.22 ± 4.80	-12.1 (-23.1 to -1.4)	**p = 0.017**
**AMDN_1_**	1.034 ± 0.30	1.028 ± 0.19	-0.6 (-9.1 to 7.9)	p = 0.886
**AMDN_2_**	2.88 ± 1.56	3.01 ± 1.06	4.5 (-9.9 to 18.1)	p = 0.515

**Table 2:** Modelling of ventilatory inhomogeneity indices of a uniformly ventilated volume using the measured V_D_/V_T _and V_T_/FRC of table 4

	**Modelling 1**	**Modelling 2**	**Difference in %**
*Model parameters*			
**V_D_/V_T_**	0.49	0.43	
**V_T_/FRC**	0.48	0.59	
			
*Inhomogeneity indices*			
**LCI**	8.16	7.67	**-6.00%**
**M_1_/M_0_**	1.79	1.61	**-10.08%**
**M_2_/M_0_**	6.48	5.48	**-15.41%**
**AMDN_1_**	0.91	0.92	**0.49%**
**AMDN_2_**	1.69	1.78	**5.66%**
